# Antagonism Versus Cooperativity with TALE Cofactors at the Base of the Functional Diversification of Hox Protein Function

**DOI:** 10.1371/journal.pgen.1003252

**Published:** 2013-02-07

**Authors:** María Luisa Rivas, Jose Manuel Espinosa-Vázquez, Nagraj Sambrani, Stephen Greig, Samir Merabet, Yacine Graba, James Castelli-Gair Hombría

**Affiliations:** 1 CABD, CSIC/JA/Universidad Pablo de Olavide, Seville, Spain; 2 IBDML, CNRS/Université de la Méditerranée, Marseille, France; 3 Akam Laboratory, University of Cambridge, Cambridge, United Kingdom; University of California San Francisco, United States of America

## Abstract

Extradenticle (Exd) and Homothorax (Hth) function as positive transcriptional cofactors of Hox proteins, helping them to bind specifically their direct targets. The posterior Hox protein Abdominal-B (Abd-B) does not require Exd/Hth to bind DNA; and, during embryogenesis, Abd-B represses *hth* and *exd* transcription. Here we show that this repression is necessary for Abd-B function, as maintained Exd/Hth expression results in transformations similar to those observed in loss-of-function Abd-B mutants. We characterize the cis regulatory module directly regulated by Abd-B in the *empty spiracles* gene and show that the Exd/Hth complex interferes with Abd-B binding to this enhancer. Our results suggest that this novel Exd/Hth function does not require the complex to bind DNA and may be mediated by direct Exd/Hth binding to the Abd-B homeodomain. Thus, in some instances, the main positive cofactor complex for anterior Hox proteins can act as a negative factor for the posterior Hox protein Abd-B. This antagonistic interaction uncovers an alternative way in which MEIS and PBC cofactors can modulate Abd-B like posterior Hox genes during development.

## Author Summary

Hox genes encode transcription factors necessary to achieve the morphological differences between anterior and posterior regions of the body. These genes have been functionally conserved during animal evolution, and similar classes can be recognized in vertebrates and invertebrates. To bind DNA and regulate many of their targets, Hox proteins interact with the MEIS and PBC transcriptional cofactors. However, this is not always the case for the most posteriorly expressed genes belonging to the Abdominal-B class. Here we show a new interaction between the Abd-B protein and these cofactors where, rather than cooperating with Abd-B, the cofactors antagonize its function. Given the conservation of the Hox proteins and their cofactors, this new mode of interaction may be also happening in other species, including vertebrates.

## Introduction

In segmented animals the differential anterior-posterior morphology is achieved during development under the control of the Hox genes [1]. Hox genes encode a conserved family of transcription factors organized in clusters in most animals. Hox clusters originated before the divergence of protostomes and deuterostomes and as a result, orthologous Hox genes can be identified between vertebrates and invertebrates that are more similar to each other than to other Hox genes in the same species [2],[3].

The development of the unique organs present in a segment is controlled by the Hox protein expressed in that segment through the regulation of specific downstream targets. In *Drosophila melanogaster* the Abdominal-B (Abd-B) protein (orthologous to Hox9/13 in mammals) induces the formation of the posterior spiracles in the eighth abdominal segment (A8) through the transcriptional activation of *empty spiracles* (*ems*), *cut* (*ct*) and *spalt* (*sal*) among other genes [4], [5]. Similarly, expression of the Sex combs reduced protein (Scr, orthologous to Hox5) in the labial segment of the head induces the formation of the salivary glands through the activation of *fork head, trachealess* and *huckebein* [6]; while expression of Ultrabithorax (Ubx) and Abdominal-A (Abd-A, both orthologous to Hox6/8) in the abdominal segments prevent the development of thoracic structures by repressing *Distalless* and *buttonhead* in the abdomen [7], [8]. The specific *in vivo* regulation of precise targets by each Hox protein contrasts with the observation that Hox proteins bind very similar DNA sequences *in vitro* [9]. In *Drosophila*, anterior and central Hox proteins (Lab, Pb, Dfd, Scr, Antp, Ubx and Abd-A [henceforth collectively referred to as anterior-Hox for simplicity]) bind TAAT sites with only the posterior-Hox Abd-B protein binding the slightly different TTAT sites [10], [11]. This *in vitro* lack of Hox DNA binding specificity is resolved *in vivo* by the use of protein cofactors that increase Hox DNA affinity and extend the binding site therefore increasing specificity for downstream target genes [12].

In *Drosophila*, the best-studied Hox cofactors are the Extradenticle (Exd) and Homothorax (Hth) proteins (homologous to the Pbx and Meis proteins in vertebrates) [13]. *In vitro* studies show that the anterior-Hox proteins bind poorly to many of their targets in the absence of Exd and Hth [14]. The Hth, Exd and Hox proteins form a trimeric complex that binds DNA with higher affinity than any of the proteins separately [15]. Exd directs the formation of the trimeric complex by binding directly to both Hth and the Hox protein. Exd can bind to various domains in the anterior-Hox proteins including the YPWM domain (present in all anterior-Hox proteins but not in the Abd-B posterior Hox proteins), the UbdA domain (present only in Ubx and Abd-A) and possibly to other domains not yet characterized [9], [16]. Hth binds Exd directly through the Homothorax-Meis (HM) domain [15] but there is no evidence of Hth binding to Hox proteins directly.

Exd translocation to the nucleus requires its binding to Hth [17]. Accordingly, Exd remains in the cytoplasm of cells that do not express Hth, while Exd is nuclear in cells expressing Hth. Moreover, in *hth* mutants Exd localization is cytoplasmic. These observations suggested a model in which Hth binding to Exd allows the translocation of Exd to the nucleus where it can bind to the Hox proteins forming the trimeric complex that binds target genes [15]. The requirement of this complex for normal Hox-target activation explains the homeotic phenotypes observed in *hth* or *exd* mutants even though they express correct levels of Hox proteins [18].

In contrast to the Exd/Hth requirement for anterior-Hox protein function, there is no clear evidence pointing to Abd-B interacting with these cofactors although such evidence exists in vertebrates for Hoxa9 protein interaction with PBX. *Abd-B* has two functions: a morphogenetic function (m) required for the formation of segment specific structures, and a regulatory function (r) that represses the transcription of anterior-Hox genes [19]. These functions correlate with the existence of two protein isoforms, which differ by the inclusion of a 5′ exon [20], [21],[22],[23]. Mutations affecting the Abd-Bm isoform result in embryos where the posterior spiracles are almost absent and the A5–A8 denticle belts resemble that in A4 indicating that Abd-Bm performs most of the morphogenetic functions [4], [19], [24]. Mutations affecting the Abd-Br isoform have minor defects in A8 but result in the formation of a small A9 denticle belt anterior to the anal pads indicating that Abd-B represses the formation of an A9 segment. However, the r isoform has some morphogenetic activity as heat shock induction of both the Abd-B m and r isoforms can induce the formation of posterior spiracles when ectopically expressed [25], [26], [27].

Contrary to the anterior-Hox proteins, addition of the Exd cofactor does not increase Abd-B's binding affinity to DNA [14]. As a result, the case for Hth and Exd interaction with Abd-B has not been studied in detail.

Here we investigate the interaction of Exd/Hth and Abd-B and find that, surprisingly, these cofactors interfere with Abd-B function during embryogenesis. We show that the presence of Exd/Hth interferes with Abd-B binding to its direct target *empty spiracles* (*ems*). This interference does not require binding of Exd/Hth to DNA and is probably achieved by Exd/Hth binding to the Abd-B homeodomain. These results uncover a novel Exd/Hth complex function and explain why in *Drosophila exd* and *hth* transcription is repressed by Abd-B protein. This novel interaction extends our understanding on the capacity of PBX MEIS proteins to modulate Hox output.

## Results

**Figure 1:**
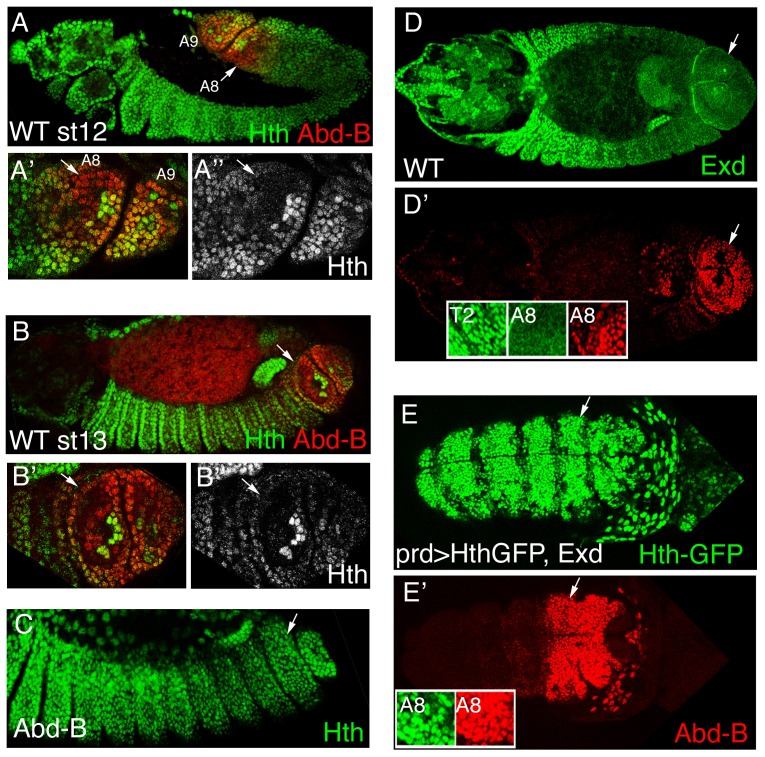
Hth and Exd downregulation by Abd-B in the posterior abdominal segments. (A–B) Hth and Abd-B expression in wild type embryos at stage 12 (A) and stage13 (B). Hth protein disappears from the dorsal area where the spiracles are formed (A′ and A″ show a close up of A rotated 180° to keep dorsal up and anterior left). Note that in the spiracle region only a cluster of 8 cells retain high Hth levels. (C) Abd-B mutant showing elevated levels of Hth in the dorsal region. (D) Wild type st14 embryo stained with anti-Exd (D, green) and anti-AbdB (D′, red). Exd protein localizes to the nucleus in anterior segments (T2 inset) while is cytoplasmic in the posterior domain where Abd-B is expressed (A8 insets). (E) Dorsal view of a st11 embryo overexpressing Exd and Hth. Insets in E′ show that although the posterior A8 segment has high levels of nuclear Exd/Hth expression (only Hth shown in E) Abd-B expression is normal (E′). White arrows point to the A8 segment. Anterior left in all panels, and dorsal up in A–C.

Despite the importance of Exd and Hth for anterior-Hox function, there is not much evidence pointing to the Abd-B proteins interacting with these cofactors. In fact, during embryogenesis *exd* and *hth* are initially expressed homogeneously along the trunk epidermis until stage 11 (st11) when their transcription is downregulated in the posterior abdominal segments [28], [29], [30]. To study more in detail the expression of Hth in the A8 and A9 segments we double stained with Abd-B antibodies and observed that Hth expression is downregulated in the dorsal region of A8 and A9 (Figure 1A–1B). This downregulation depends on Abd-B function as dorsal levels of Hth are restored in Abd-B mutant embryos (Figure 1C). In this region it had been described that Abd-B excludes Exd protein from the nucleus [[28] and Figure 1D′ insets] probably because its effect on Hth expression.

### Coexpression of Hth and Exd hampers Abd-B morphogenetic capacity

**Figure 2:**
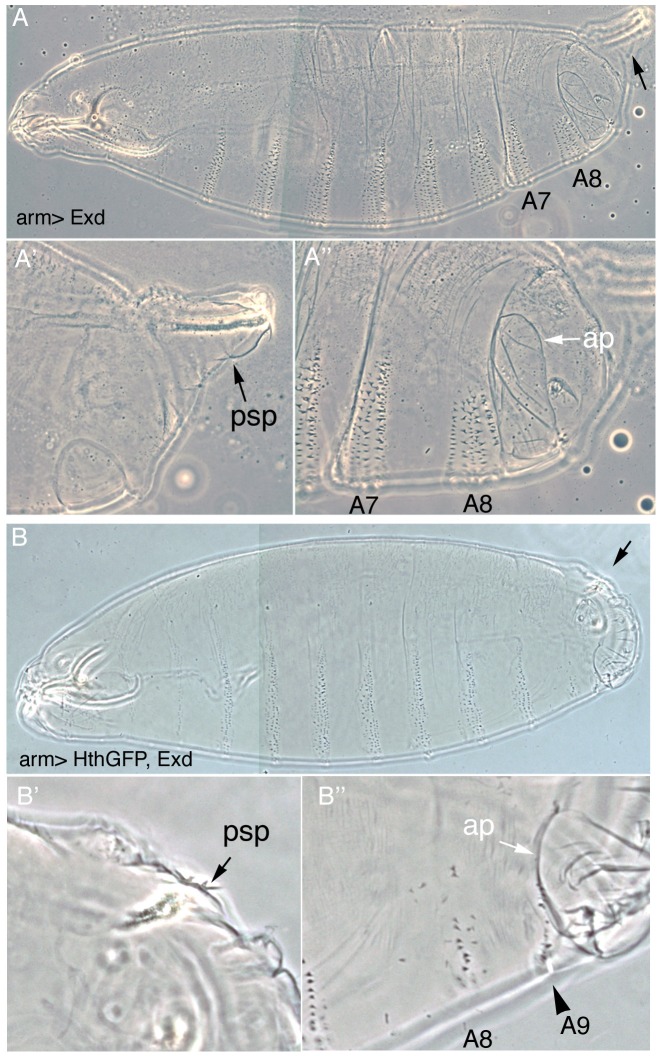
Downregulation of Exd/Hth expression is required for the normal development of the posterior abdominal segments. (A) Ectopic expression of Exd results in viable larvae with normal posterior spiracles (psp, black arrow A′) and a normal denticle pattern with the A8 denticle belt immediately abutting the anal pads (ap, white arrow A″). (B) Simultaneous ectopic expression of Exd/Hth results in larvae that form aberrant posterior spiracles (B′), a reduced A8 denticle belt and an extra A9 belt anterior to the anal pad (B″).

To test if the downregulation of *exd* and *hth* observed in wild type embryos is required for the normal development of the A8 segment we artificially maintained their expression using the Gal4 system. Expression of Exd in the ectoderm using the *arm-Gal4* or the *69B-Gal4* lines driving *UAS-exd* results in embryos with normal cuticles (Figure 2A) and the same is true for *UAS-hth* (not shown). In contrast, coexpression of Exd and Hth gives rise to larvae with abnormal posterior spiracles and a reduced A8 denticle belt (Figure 2B–2B′). Interestingly, in many embryos a small A9 denticle belt forms (Figure 2B″), a phenotype also observed in the *Abd-B^Uab-1^* and *Abd-B^UabX23-1^* loss-of-function alleles [19]. As these phenotypes could be caused by abnormal Abd-B expression, we stained embryos expressing ectopically both cofactors with anti-Abd-B. Using several Gal4 lines we observed that Abd-B localization in cells ectopically expressing Exd/Hth is normal (Figure 1E–1E′) showing that the transformations caused in the posterior segments are not due to altered Abd-B expression.

**Figure 3:**
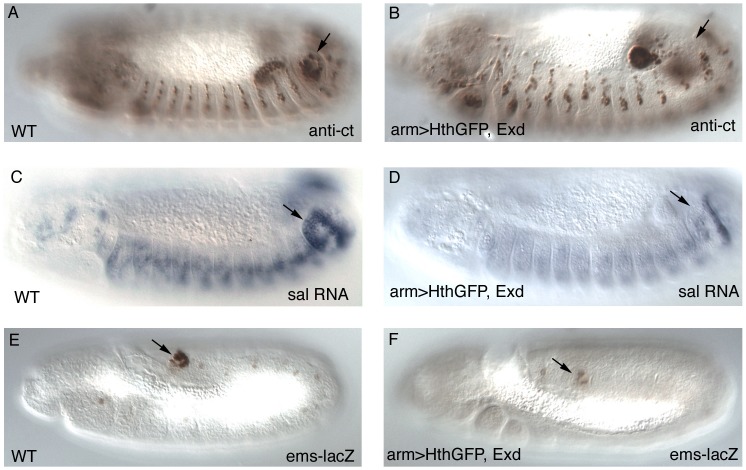
Expression of early Abd-B downstream targets after Exd/Hth ectopic induction. Expression of Cut protein (A–B), *spalt* RNA (C–D) and the *ems* posterior spiracle reporter gene (E–F) in wild type (A,C,E) or embryos expressing ectopically Hth and Exd with the *arm-Gal4* line (B, D, F). Arrows point to the posterior spiracle site. Embryos in (A–D) have retracted the germ band while those in (E–F) are at extended germ band, and are thus folded with the A8 segment close to the head.

To find out at what level of the Abd-B genetic cascade the spiracle defects are caused we analyzed the expression of the early Abd-B targets [5]. We observe that the expression of *ct* and *sal* is downregulated in these embryos (Figure 3A–3D) suggesting that overexpression of Exd/Hth interferes with the normal activation of Abd-B downstream targets. The *ems* gene is required for spiracle development and its expression in the posterior spiracles is regulated by an enhancer that depends on *Abd-B* function [25]. We observe that expression of the *ems* spiracle enhancer is also downregulated in embryos overexpressing Exd/Hth (Figure 3E–3F). Taken together, these results suggest that in the presence of the Exd/Hth complex Abd-B proteins are less efficient in the activation of their direct targets. These results indicate that although Exd/Hth are positive cofactors of anterior-Hox proteins, they may also have a previously unnoticed negative effect on Abd-B function.

### Exd/Hth affect the function of both Abd-B isoforms

**Figure 4:**
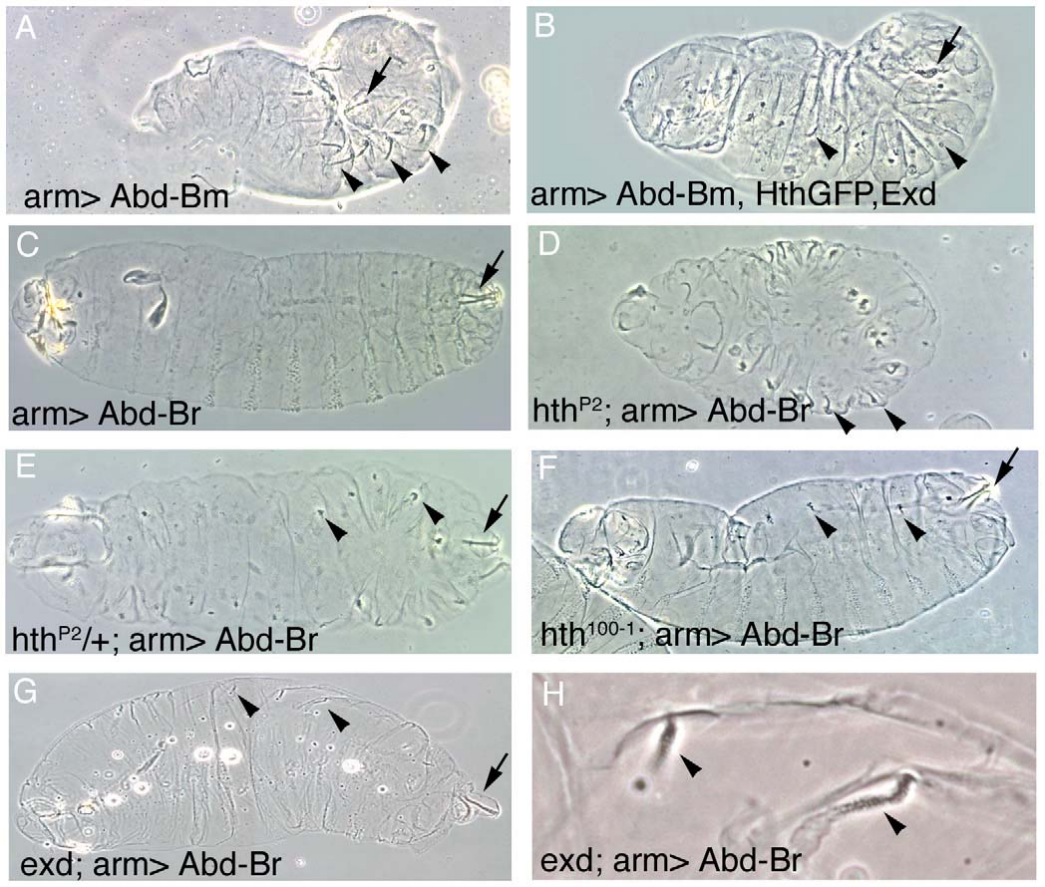
Effect of the ectopic expression of Abd-B isoforms on the development of embryos expressing different levels of Hth and Exd. (A) Abd-Bm ectopic expression with the *arm-Gal4* line induces posterior spiracles in ectopic positions (In this figure arrows point to normal A8 posterior spiracles and arrowheads to ectopic spiracles). (B) Ectopic expression of Abd-Bm can only form small remnants of posterior spiracles when co-expressed with the Hth and Exd proteins. (C) Abd-Br ectopic expression with the *arm-Gal4* line is not capable of inducing ectopic posterior spiracles in a wild type background. (D) Abd-Br can induce ectopic posterior spiracles in *hth^P2^* null embryos. (E) Abd-Br can induce small ectopic posterior spiracles in *hth^P2^* heterozygous embryos. Note that the ectopic spiracles in the posterior segments are more complete. (F) In hypomorphic *hth^100-1^* alleles lacking the homeodomain containing isoform but still expressing the homeodomainless protein Abd-Br is not capable of efficiently inducing ectopic posterior spiracles (compare to panel D). These embryos have tiny spiracles that can be explained by the reduction of total Hth protein caused by this allele, resulting in levels more similar to those present in heterozygous *hth^P2^* embryos shown in panel E. (G) Abd-Br expression in zygotic *exd^YO12^* mutant embryos induces ectopic spiracles. (H) Close up of (G) showing some ectopic spiracles. All experiments in this figure were performed at 25°C.

To test if Exd/Hth expression affects the function of both Abd-B isoforms, we first studied how the phenotypes obtained after ectopically expressing Abd-Bm are affected by the simultaneous expression of Exd/Hth. As previously reported with other Gal4 drivers [31], ectopic expression in the ectoderm of UAS-Abd-Bm with *arm-Gal4* causes the formation of ectopic posterior spiracles in all trunk segments (Figure 4A) and the same is true if Abd-Bm is coexpressed with two irrelevant UAS constructs (see materials and methods). In contrast, simultaneous expression of Hth and Exd with Abd-Bm severely reduces the length of the ectopic spiracles (Figure 4B) confirming that Abd-Bm cannot fully function in the presence of these Hox cofactors.

Ectopic expression of the Abd-Br isoform with *arm-Gal4* does not induce ectopic spiracles (Figure 4C) despite the fact that antibody stainings indicate that the protein is expressed at high levels (Figure S1A–S1C). This is probably due to Abd-Br having an inefficient morphogenetic function, as using stronger Gal4 lines or increasing the expression levels of Abd-Br by performing the experiment at 29°C, a temperature favouring Gal4 activity, results in the formation of ectopic posterior spiracles (Figure S1D–S1E). This confirms previous experiments using heat shock inducible constructs that demonstrated that both the Abd-Bm and r isoforms perform the morphogenetic function albeit Abd-Br is less efficient [25], [26], [27].

To test if Abd-Br is also competed by Exd/Hth we took advantage of the weak morphogenetic capacity shown by Abd-Br at 25°C (Figure 4C) and studied how varying the levels of endogenous Exd or Hth affects its function. Ectopic expression of Abd-Br in a *hth* homozygous mutant background induces ectopic spiracles similar to what Abd-Bm does in a wild type background (Figure 4 compare 4D to 4A), indicating that endogenous Hth can partially block Abd-Br activity. This effect is dependent on Hth protein concentration, as in heterozygous *hth/+* embryos, expression of Abd-Br at 25°C can induce small ectopic spiracles (Figure 4E). We also observe that the ectopic spiracles appear to be more complete in the A2–A7 segments where antibody stainings show there are lower levels of endogenous Hth protein. Similar to *hth* mutants, ectopic Abd-Br expression in *exd* zygotic mutants results in the formation of ectopic spiracles (Figure 4G–4H). These spiracles are smaller than those observed in a *hth* mutant background probably due to the maternal *exd* contribution. Taken together, the above results show that the function of both Abd-B isoforms is sensitive to the Exd/Hth protein levels.

### Molecular characterization of a minimal posterior spiracle Abd-B regulated enhancer

Abd-B has been suggested to control directly *ems* transcription in the spiracle through an enhancer located in a 1.2 kb region upstream of the promoter but the Abd-B binding sites mediating this interaction have not been identified [25]. As we found that the *ems* spiracle enhancer is downregulated in embryos where Exd/Hth expression is maintained (Figure 3E–3F), we decided to confirm its direct regulation by Abd-B and study how Exd/Hth can affect its expression.

Subdivision of the 1.2 kb fragment shows that the central 0.35 kb element is responsible for spiracle expression (Figure S2A–S2D). The 0.35 kb element is regulated by Abd-B and behaves like the original 1.2 kb fragment, responding to ectopic Abd-B expression (Figure S2E) and losing its expression in Abd-B null mutants (Figure S2F). Further reduction of the 350 bp element from the 5′ or the 3′ end abolishes spiracle expression (Figure S2I–S2L). This 350 bp element contains six putative Abd-B binding sites (TTAT) five of which are conserved in twelve *Drosophila* species analyzed (Figure S3, red boxes). Chromatin Immunoprecipitation (ChIP) in S2 cells transfected with HA-tagged Abd-B shows that Abd-B can bind the *ems* posterior spiracle enhancer *in vivo* (Figure S2G).

**Figure 5:**
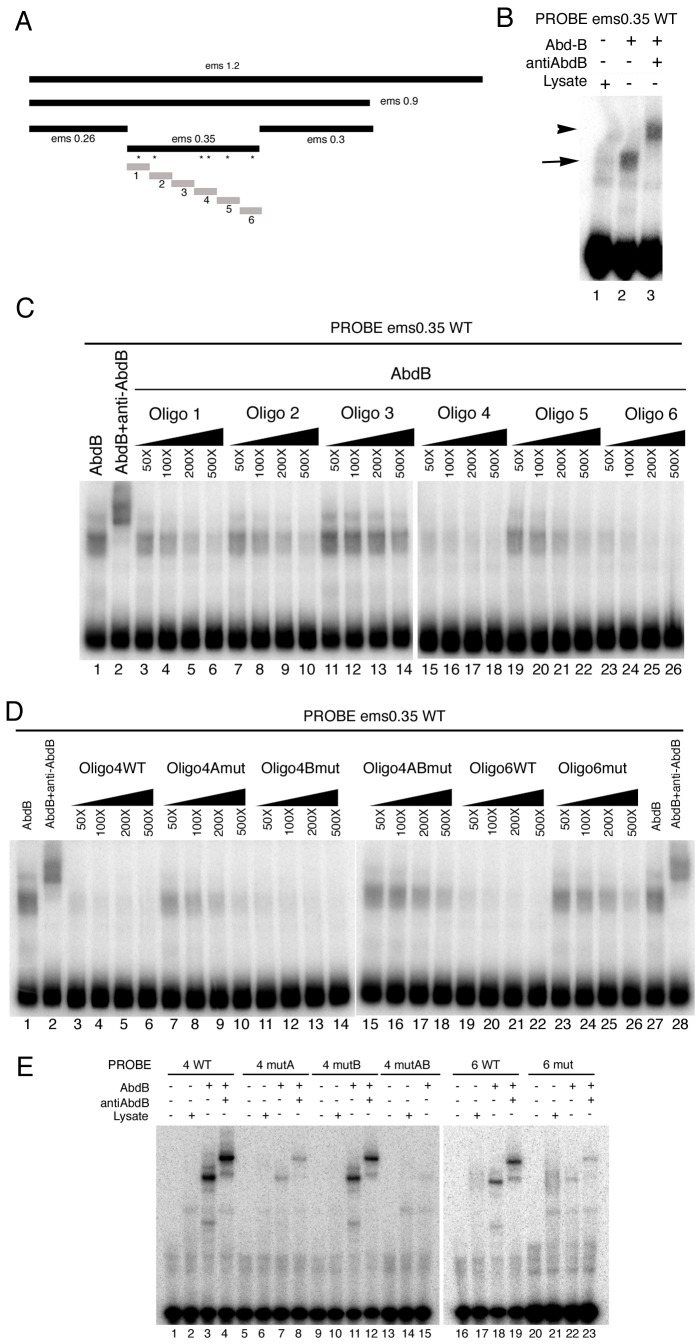
Characterization of the Abd-B binding sites in the *ems* posterior spiracle enhancer. (A) Dissection of the *ems1.2* posterior spiracle enhancer. Black bars represent fragments tested in transgenic *lacZ* constructs. Grey bars represent oligos tested in EMSA. Asterisks indicate the location of putative Abd-B binding sites in the *ems0.35* fragment. (B) EMSA showing Abd-B binding to the *ems0.35* enhancer (lane 2 arrow), this band is supershifted by anti-AbdB confirming that the complex contains Abd-B (lane 3 arrowhead). (C) Abd-B binding to the *ems0.35* enhancer is competed by cold oligos containing Abd-B sites. Oligos 4 and 6 show higher affinity for Abd-B. Oligo 3 that does not contain putative Abd-B sites does not compete (lanes 11–14). (D) EMSA showing that wild type cold oligo 4 (lanes 3–6) and oligo 6 (lanes 19–22) can compete for Abd-B binding to the *ems0.35* enhancer while cold oligo 4 with a mutation on both 4A and 4B sites (lanes 15–18) or oligo 6 with a mutation on its only site (lanes 23–26) cannot compete Abd-B binding. Note that separately mutating in oligo 4 site 4A (lanes 7–10) or site 4B (lanes 11–14) shows that site 4A has higher affinity for Abd-B. However, comparison of the independent mutations to the double 4A4B mutant suggests both sites are functional (lanes 15–18). Triangles in panels C and D represent increasing amounts of the indicated cold oligo competitor. (E) EMSA showing that Abd-B binds to wild type oligos 4 and 6 through the predicted putative Abd-B sites, as binding to the oligos decreases when these sites are mutated (compare WT lanes 3–4 and 18–19 with lanes labelled as mut). Site 4A and 6 bind to Abd-B with higher affinity than site 4B.

Electrophoresis mobility shift assays (EMSA) confirm the binding of Abd-B to the 350 bp element (Figure 5B). To test if all putative Abd-B sites in the 350 fragment are bound by Abd-B with equal affinity we made six similar sized oligos covering the whole fragment (Figure 5A grey boxes) and tested their capacity to compete for Abd-B binding to the whole 0.35 fragment. At high concentration all oligos, except oligo three that does not contain predicted Abd-B binding sites, can compete for Abd-B binding (Figure 5C). However, at lower concentrations only oligo 4 and oligo 6 are able to compete efficiently (Figure 5C) indicating that these sites have higher affinity for Abd-B.

To confirm that Abd-B binds the oligos through the predicted sites, we mutated the TTAT sites in oligos 4 and 6, and analyzed the capacity of Abd-B to bind these oligos in EMSA. Mutation of both putative binding sites in oligo 4 abolishes Abd-B binding to it (Figure 5E compare lane 3 with 15); with mutation of site 4A (lane 7) having a stronger effect than mutation of site 4B (lane 11) when mutated independently. Similarly, mutation of the putative binding site in oligo 6 strongly decreases its ability to be bound by Abd-B in EMSA (Figure 5E compare lane 18 with 22). This confirms that Abd-B binds to the predicted sites, and shows that *in vitro* each site binds Abd-B with different affinities.

We next tested the capacity of mutant and wild-type cold oligos 4 and 6 to compete the *ems0.35* fragment for Abd-B binding. As expected, even at the high concentration, oligos with mutant Abd-B sites cannot compete for binding (Figure 5D). Mutation of the only putative Abd-B binding site in oligo 6 almost abolishes its ability to compete (Figure 5D compare lanes 19–22 with 23–26). Mutation of both putative binding sites in oligo 4 almost abolishes Abd-B binding (Figure 5D lanes 3–6 compared with 15–18); again with site 4A (lanes 7–10) having more effect than site 4B (lanes 11–14) when mutated independently.

**Figure 6:**
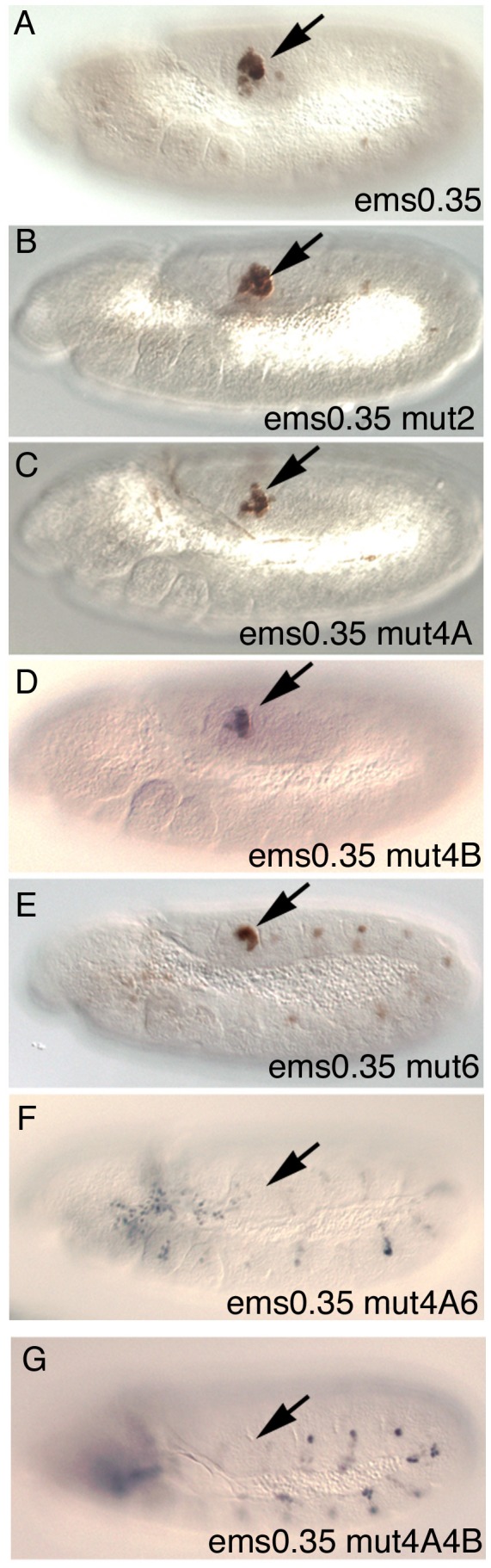
Expression of different *ems0.35* enhancer variants in st11 embryos. (A) ßGal expression of the *ems0.35* wild type enhancer. (B) Expression of *ems0.35* with a mutation on the second putative Abd-B site. (C–E) Reduced expression of *ems0.35* constructs carrying a single mutation on either the 4A (C), 4B (D) or 6 (E) putative Abd-B sites. (F) Expression of *ems0.35* double mutant in site 4A and 6. (G) *ems0.35* constructs double mutant for site 4A and 4B show no spiracle expression. Arrows point to the posterior spiracle primordium.

To test their *in vivo* requirement, we mutated single Abd-B sites in the *ems0.35* enhancer. While mutation of site 1 or site 2 does not affect spiracle expression noticeably (Figure 6B and Figure S2H), single mutation of putative sites 4A, 4B or 6 slightly reduces expression (Figure 6C–6E). Simultaneous mutation in *ems0.35* of sites 4A and 6 strongly reduces spiracle expression (Figure 6F) with only occasional spiracles having residual expression; while mutation of sites 4A and 4B completely abolishes spiracle expression in all embryos (Figure 6G). These results are consistent with Abd-B controlling the expression of the *ems* spiracle enhancer by binding to several sites in an additive manner. These experiments and the deletion series show that sites 4A, 4B and 6 are necessary but not sufficient for spiracle expression, as fragments D and E that do not affect these sites also lose spiracle expression (Figure S2J, S2L).

### Abd-B binding to its target DNA is competed by Exd/Hth

**Figure 7:**
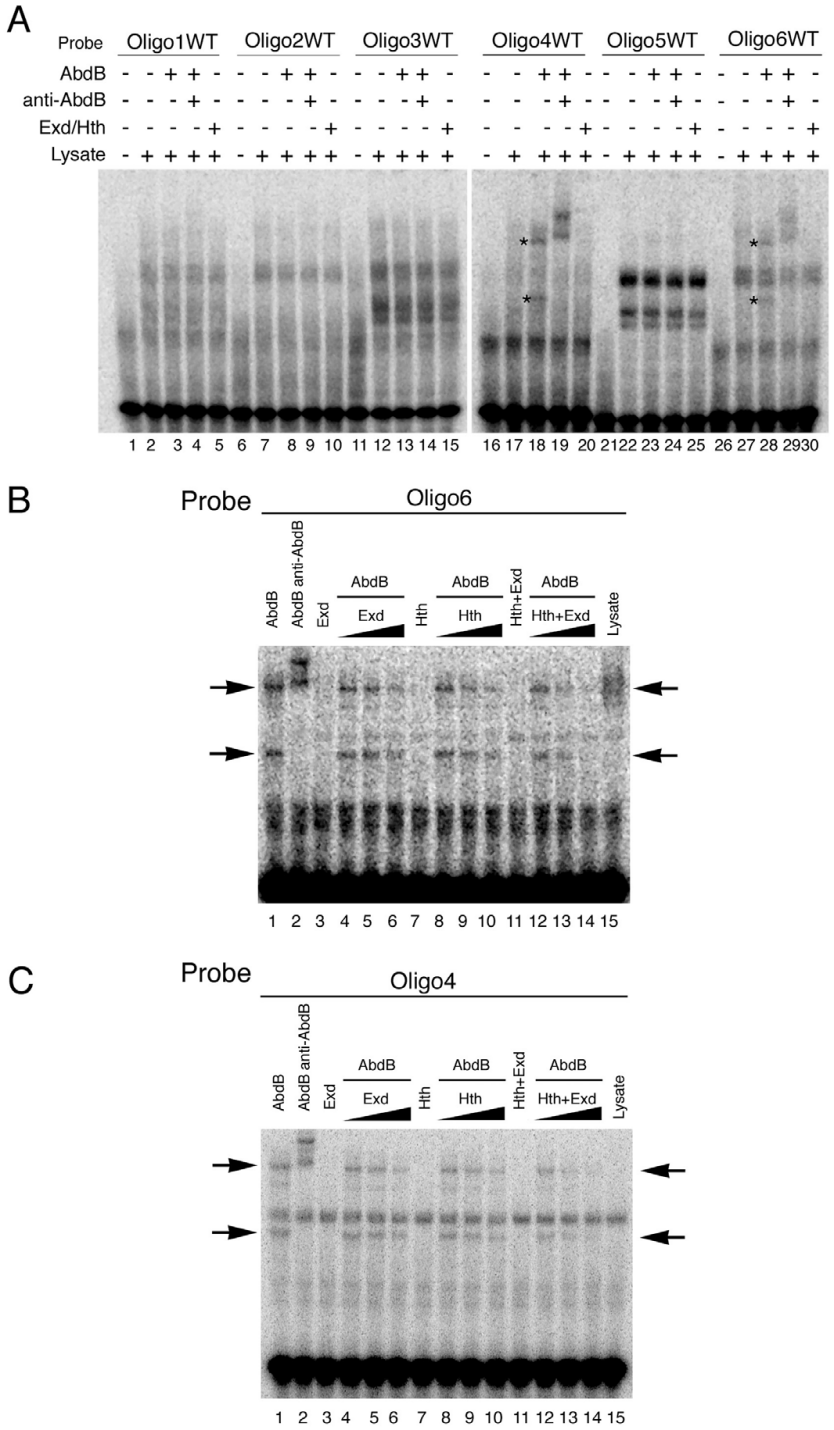
Exd/Hth interfere with Abd-B binding to the *ems* spiracle enhancer. (A) EMSA showing that Exd/Hth does not bind to the *ems0.35* oligos (lanes 5,10,15,20,25,30) in conditions where Abd-B binds to oligos 4 and 6 (lanes 18–19 and 28–29, asterisks). (B–C) EMSA showing that Abd-B binding to oligos 4 and 6 is partially competed by increasing amounts of Exd/Hth proteins (lanes 12–14). Separate expression of Hth or Exd has only a small effect on Abd-B binding to these oligos.

To understand how Exd/Hth compete Abd-B activation of *ems* we first analyzed the capacity of these cofactors to bind the *ems* spiracle enhancer. In EMSA experiments we could not detect Exd/Hth binding to any of the six *ems* oligos (Figure 7A lanes 5,10,15,20,25,30) in conditions where we could detect Abd-B binding to oligos 4 and 6 (Figure 7A lanes 18,28 asterisks).

We next analyzed the effect of adding Exd, Hth or Exd/Hth to oligo 6 where the Abd-B site overlaps a predicted Exd/Hth site (Figure S3). Separate addition of Exd or Hth has a small effect on Abd-B binding to the DNA, while adding simultaneously Exd/Hth decreases the affinity of Abd-B for oligo 6 in a concentration dependent manner (Figure 7B lanes 12–14). Interestingly, adding Exd/Hth to oligo 4 that does not contain any predicted Exd/Hth sites also interferes with Abd-B binding (Figure 7C lanes 12–14) as efficiently as with oligo 6 where the predicted Abd-B Exd/Hth binding sites overlap. These results suggest that Exd/Hth interference with Abd-B is not due to competition for occupancy of overlapping binding sites.

### A Hth homeodomainless protein can interfere Abd-B function

As Exd/Hth does not bind oligos 4 and 6 *in vitro*, it is possible that interference with Abd-B binding to DNA is not due to competition for DNA binding but due to direct binding of Abd-B to the Exd/Hth complex.

In the embryo, there are several naturally expressed Hth isoforms. Some isoforms contain the DNA binding homeodomain, while others lack the homeodomain but still include the HM domain [32]. To test if Hth proteins without the homeodomain are capable of competing Abd-B function *in vivo*, we studied the *hth^100-1^* allele that only affects the homeodomain containing isoform [32]. In *hth^100-1^* embryos, ectopic expression of UAS-AbdBr at 25°C does not form well-developed ectopic spiracles as those formed in *hth^P2^* alleles (compare Figure 4F and 4D), indicating that the homeodomainless Hth isoform can compete Abd-Br morphogenetic function in vivo. However, in these embryos some small spiracle structures are formed not seen in a wild type background (compare Figure 4F and 4C), suggesting that although the homeodomain of Hth is not strictly necessary, the full isoform competes Abd-Br posterior spiracle morphogenetic function more efficiently.

### Abd-B protein domains required for Exd/Hth functional antagonism

**Figure 8:**
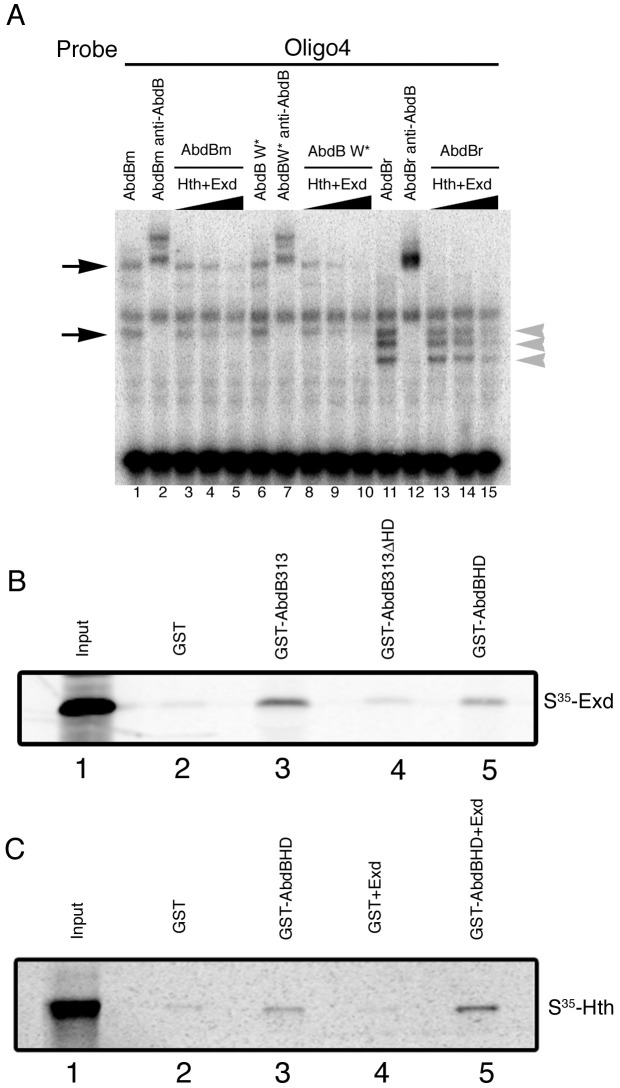
Direct Abd-B homeodomain binding to the Exd/Hth complex. (A) EMSA showing that both Abd-Bm (lanes 3–5) and Abd-Br (lanes13–15) binding for oligo 4 is competed in the presence of Exd/Hth. Similar competition is observed over an Abd-B variant with a conserved W residue mutated (lanes 8–10). Note that in each lane several size bands appear (black arrows and grey arrowheads in panel A). These bands are specific as they are supershifted by anti-AbdB. We interpret them as due to Abd-B being translated *in vitro* from internal methionines as the smaller band in lane 1 coincides with the larger Abd-Br band in lane 11. (B–C) GST-Abd-B pull-down experiments with Exd and Hth. Beads binding GST or GST fused to the Abd-B C-terminal fragments were incubated with methionine-S^35^ labelled Exd (B) or Hth (C). (B) An Abd-B C-terminal fragment binds S^35^-Exd. This interaction is reduced when the homeodomain is deleted from the fragment, and the Abd-B homeodomain by itself can bind Exd. (C) Abd-B homeodomain only weakly binds S^35^-Hth (GST-AbdBHD, third lane), but the interaction is enhanced by the presence of unlabelled Exd protein (GST-AbdBHD+Exd, fifth lane) indicating the formation of a trimeric complex. For each of the S^35^-labelled proteins, 25% of the amount used in the binding reactions was directly loaded in the first lanes (Input).

Exd binds anterior Hox proteins trough several domains, among them the YPWM domain. Although Abd-B lacks this element, many Abd-B like proteins contain at a similar position with respect to the homeodomain a conserved tryptophan (W) amino acid [33]. To investigate the possibility that Abd-B and Exd/Hth interact through this amino acid we analyzed the capacity of Exd/Hth to interfere with an Abd-B protein where this tryptophan residue has been mutated to Alanine (Abd-B W*). As shown in Figure 8A (lanes 8–10), mutation of this tryptophan does not prevent Exd/Hth interference with Abd-Bm DNA binding.

To investigate if the competitive interaction requires a particular Abd-B protein domain, we analyzed if the competition occurs with both Abd-B isoforms. We observe that adding Exd/Hth interferes with both Abd-Bm and Abd-Br isoforms binding to oligo 4 (Figure 8A lanes 3–5 and 13–15). In these experiments we observe the formation of different size bands (Figure 8A lane 1 black arrows and lane 11 grey arrowheads). These bands are specific as they are supershifted by anti-AbdB (Figure 8A lanes 2 and 12). As the smaller Abd-Bm band in lane 1 coincides with the larger Abd-Br band in lane 11 we interpret these bands as being the result of *in vitro* translation from internal Abd-B methionines. The observation that Exd/Hth can compete with even the smallest Abd-Br fragment suggests that the interference is due to interaction with the C-terminal end of Abd-B where the homeodomain is located.

To find out if Exd or Hth interact directly with the Abd-B C-terminal region we performed GST pull-down experiments. We observed that Exd interacts with GST fused to Abd-B313, a C-terminal fragment including the last 181a.a (from 313 to 493) (Figure 8B, lane 3). We observed that Exd interaction with this fragment is reduced if we remove the homeodomain (AbdB313ΔHD, lane 4) and found that Exd can interact directly with the Abd-B HD fragment (lane 5). In contrast, GST fused to the Abd-B homeodomain has a weak interaction with Hth (Figure 8C lane 3). However, when cold Exd is added to the mixture higher levels of Hth are isolated (Figure 8C lane 5). These experiments show that in the absence of DNA, the Exd/Hth complex can bind the Abd-B homeodomain, providing a mechanistic explanation for the observed in vivo antagonistic effect between these proteins.

## Discussion

The evolution of Hox proteins was fundamental for the acquisition of morphological differences in the antero-posterior axis of animals. Comparison between all extant animals indicates that the difference between posterior (Abd-B like) and anterior-Hox genes occurred early in evolution. This happened before the Hox and ParaHox clusters diverged, in what has been called a protoHox cluster [34]. This early divergence has resulted in Abd-B having a different character to all other anterior-Hox proteins, with the most striking difference being Abd-B binding to a TTAT DNA core site [and TTAC with lower affinity [35]] while other Hox proteins bind to a TAAT core [10], [11]. The divergence is also reflected at the protein sequence level with all anterior-Hox proteins having a YPWM motif that is absent or highly reduced from Abd-B like proteins [3], [33]. At the functional level, a major difference is the use anterior-Hox proteins do of the Exd/Hth complex as a positive cofactor to increase target DNA-binding efficiency, while Abd-B does not require it [14], [18]. Here we have shown *in vivo* and *in vitro* a new relationship between Abd-B and Exd/Hth where these positive Hox cofactors can also have an antagonistic interaction with Abd-B protein function, providing an explanation to why Abd-B represses the transcription of *exd* and *hth* genes during development.

### Abd-B activates *ems* transcription through the action of additive binding sites

The *ems* gene had been suggested to be a direct target of Abd-B in the posterior spiracles [25]. Despite being one of the first putative Hox targets analyzed, the lack of direct mutational evidence has resulted in *ems* being excluded from most Hox-target compilations [1], [9]. Here we have trimmed down this element to 350 bp and demonstrated that the spiracle enhancer is directly activated by Abd-B. This enhancer contains several sites with different Abd-B affinities all of which conform to the TTAT core sequence. Mutation of single sites does not eliminate enhancer expression, while simultaneous mutation of two high affinity sites abolishes the *in vivo* enhancer function. This suggests that similarly to *yellow* and *bric-a-brac*, two confirmed Abd-B direct targets analyzed to date [35], [36], Abd-B sites act additively. Our observation that mutating the low affinity Abd-B binding site 1 has no effect on the *ems* spiracle enhancer expression, while the deletion of the element abolishes expression (Figure S2H, S2L) indicates the presence of binding sites for cofactor or collaborator proteins acting in concert with Abd-B to achieve intrasegmental specificity.

### Exd/Hth as a competitor of Abd-B transcriptional activation

Although additional bona fide targets should be analyzed to test how general is Exd/Hth competition on Abd-B function, our results indicate that this may be widespread during embryogenesis. We have found that induction of Exd/Hth in the A8–A9 segments not only affects the posterior spiracles, but also perturbs ectodermal cuticular structures controlled by Abd-B as well as downregulates the expression of the Abd-B early spiracle targets analyzed [37]. Moreover, it was described that the ectopic expression of Hth in the *Drosophila melanogaster* male abdomen causes a lack of pigmentation [15]. As it has been found that Abd-B induces male abdominal pigmentation by activating transcription of the *yellow* gene in the A5 and A6 segments [36], the effect of Hth expression on male pigmentation could also be explained by Exd/Hth interfering during larval development with the activation of *yellow* by Abd-B. Similarly, in the accompanying paper, Graba and collaborators [38] show that *Dll* repression by Abd-B in the posterior abdominal segments is also competed by Hth activation. This differs from our results as we only observe effects when both Exd and Hth are expressed in vivo. The difference may be explained as due to certain targets being more sensitive than others to the competition. In fact, our in vitro experiments show that Hth can bind weakly to Abd-B, and that this binding is increased by the addition of Exd (Figure 8C). The effect on Abd-B function, rather than a competition for binding sites in each specific target, could be due to a blocking interaction of Exd/Hth on Abd-B a possibility that is suggested by the direct binding we observe between Abd-B and the Exd/Hth complex. This is also supported by our observation that binding of Abd-B to oligo 4 is competed by Exd/Hth despite the absence of putative binding sites for these cofactors on this element. The direct interaction of Exd/Hth with the Abd-B homeodomain offers a plausible explanation for the observed antagonistic effect that Exd/Hth causes *in vivo* and *in vitro*.

### How general is Exd/Hth competition for Abd-B?

Despite the many instances where we show competition between Abd-B and Exd/Hth during embryogenesis, there is at least one important case where the competition does not seem to happen, and this is the regulation of *hth* and *exd* transcription itself. Maternal and zygotic Exd and Hth proteins are expressed homogeneously along the antero-posterior axis until extended germ band (st11) when posterior Hox proteins downregulate their expression in the posterior abdomen [28], [29], [30]. Thus, at least in this case, the presence of Exd/Hth is incapable of blocking the Abd-B repressive function on *hth* transcription on the dorsal side of A8 and A9. Why competition does not occur on *hth* downregulation during this stage of embryogenesis is unclear. A simple explanation could be that although Abd-B function is also competed by the presence of Exd/Hth, Abd-B's maintained expression will eventually overturn the blocking effect of the Exd/Hth protein therefore repressing *exd* and *hth* transcription. Alternatively, we cannot discard the existence of a dedicated factor expressed at this stage preventing the competition of Exd/Hth with Abd-B. The expression of such factor in some cells but not in others would explain why Abd-B represses Hth in only some but not all cells of A8 and A9. The existence of this additional factor could also explain the surprising observation that some cells in the Abd-B domain have nuclear Hth without corresponding nuclear Exd. Our results open up the possibility of the existence of a dual Hth/Exd interaction with Abd-B: the antagonistic interaction we uncover here and, a different one, where Abd-B may not be competed by Exd/Hth and in fact could be acting as a positive cofactor as it happens with more anterior Hox genes. This may be happening in the genital discs where both Exd/Hth and Abd-B are co-expressed [39].

### Possible function of the Abd-B Exd/Hth competitive interactions

No cofactors have yet been identified for the Abd-B protein. The finding that the main positive cofactor of the anterior-Hox proteins is a competitor for the posterior Hox proteins is interesting. It is well established that Abd-B represses anterior-Hox gene transcription [40]. The fact that it also represses the positive cofactors of anterior-Hox proteins reinforces the prevalence of Abd-B expression and function in posterior segments. Our finding that not only Exd/Hth reinforces anterior Hox function but also counteracts Abd-B function uncovers a complementary mechanism for the stabilization of the anterior vs posterior segment information, where any accidental ectopic Abd-B expression in anterior segments would be quickly dampened down by the presence of the Exd/Hth complex before it has had a significant transcriptional effect on the repression of anterior *Hox* genes or on *hth* and *exd* transcription.

Another important function could be in cells where Abd-B and anterior-Hox proteins are coexpressed. Although the negative cross-regulatory interactions between Hox genes in *Drosophila* results in most cells expressing either an anterior or a posterior Hox protein [40], in the central nervous system or the ventral ectoderm of the embryo there are well documented cases where both proteins are coexpressed. This is illustrated by the dMP2 and MP1 pioneer neurons in the central nerve cord [41], or by the A8 segment that requires both Abd-A and Abd-B function to shape the denticle belt [24]. It is easy to imagine that in cells where both anterior-Hox proteins and Abd-B are coexpressed, the levels of Exd/Hth complex present can modulate the transcriptional output favouring either the function of one or the other Hox protein. In addition Abd-B repression of *exd* and *hth* transcription would limit the targets Abd-A could activate to those bound with high affinity in the absence of the cofactors as it has been found for Ubx in the distal part of the appendage (haltere) [42].

### Evidence for Pbx/Meis competing posterior Abd-B like Hox in vertebrates

An open question is to what extent a similar interference also happens in mammals where the Hox proteins have expanded to 39 orthologs and multiple MEIS and PBX proteins exist [2], [13], [43]. In vertebrates there is evidence of Pbx1 binding to posterior Abd-B like Hox proteins. HoxA9-Pbx1 crystal structure showed that the conserved W amino acid present in HoxA9 at a position homologous to the YPWM sequence interacts with Pbx-1 [44]. HoxB9 and HoxA10 that posses this conserved W increase their DNA binding affinity in the presence of Pbx-1 in a similar manner as what happens with anterior-Hox proteins [33]. In contrast, Pbx1 does not increase the affinity to DNA of HoxA11, HoxD12 and HoxD13, which lack this W amino acid [33]. In fact, observation of the published results suggest that some competition to DNA binding similar to what we observe with Abd-B in *Drosophila* may happen in vertebrates (see Figure 1A in [33]).

Several papers have reported detailed analysis of the molecular interaction of PBX/MEIS proteins with either HoxA9 or HoxA10. Similar to our findings in *Drosophila*, addition of increasing amounts of MEIS leads to a decrease of HoxA9 or Pbx/Hoxa9 binding to the DNA (see Figure 2 in [45]). Although in this work the authors observed the formation of a trimeric complex on DNA that we have failed to detect, binding of the trimeric complex to the promoter was unable to increase transcription [45].

More recently, it has been reported that during osteoblastogenesis Pbx1 negatively regulates HoxA10 mediated transcription [46]. Although both results coincide with our observations in *Drosophila* where Exd/Hth compete instead of collaborating with Abd-B, there is one case where PBX1a and MEIS1b interact with HoxA10 as positive cofactors in the transcriptional regulation of p21 [47]. Thus, although further experiments should be done in *Drosophila* and vertebrates to clarify if there is a dual function of Exd/Hth and Pbx/Meis on Abd-B like Hox proteins, we believe that the existing results are indicative of a novel antagonistic function that contrasts with their well known cooperative effect with anterior-Hox proteins. Our in vivo observations indicating the existence of antagonistic interactions and recent results showing that, in vitro, Hth/Exd interaction with Abd-B transforms the unique DNA binding specificity of Abd-B from TTAT to that of a more anterior Hox gene [48] show the enormous modulatory potential that these cofactors can have on the Abd-B like Hox protein output.

## Materials and Methods

### Fly strains and crosses

The following Gal4 driver and UAS lines were used: *arm-Gal4*, *69B-Gal4*, *prd-Gal4*, *nullo-Gal4*, *UAS-hth-gfp* (isoform containing both the HM and homeodomain), *UAS-exd*, *UAS-Abd-Bm*, *UAS-y*, *UAS-t*. We used the *hth^P2^* (null mutant affecting all isoforms), *hth^101-1^* (mutant only affecting the homeodomain containing isoform), *exd^YO12^*, and the *Abd-B* loss-of-function allele *Uab^X23-1^* affecting only the Abd-Br function. The *ems1.2-lacZ* reporter line was a gift from Bill McGinnis [25].

To test the interference of Exd/Hth with Abd-Bm function we crossed homozygous *arm-Gal4* males to *UAS-exd; UAS-AbdBm; UAS-hth-GFP e/TM6B* females. As a control we crossed the *arm-Gal4* males to *w; UAS-AbdBm; UAS-y, UAS-t/TM6B* females. In both crosses we expect at least 50% of the embryos to have well developed ectopic spiracles due to the expression of *UAS-Abd-Bm* and absence of the two accompanying UAS constructs (either *UAS-exd, UAS-hth* in experimental or *UAS-y, UAS-t* in control embryos). We observed that in the cross generating *arm-Gal4; UAS-exd; UAS-Abd-Bm; UAS-hth-GFP* embryos, 54.7% of them had well developed spiracles and the rest formed small and medium spiracles as those shown in Figure 4B (n = 86). In the control cross generating *arm-Gal4; UAS-Abd-Bm; UAS-y, UAS-t* embryos, 85,4% had well developed ectopic spiracle formation (n = 76). These results indicate that coexpression of *UAS-exd UAS-hth* strongly reduces the effect of *UAS-Abd-Bm* expression while *UAS-y UAS-t* does not.

### Antibodies and RNA in situ probes

Anti-Exd and anti-Hth (Kindly donated by R. Mann and N. Azpiazu); anti-AbdB 1A2E and anti-ct 2B10 (Hybridoma Bank); and anti-ßGal mouse (Promega) primary antibodies were used. For *sal in situ* we used an antisense RNA probe.

### Constructs

The *UAS-Abd-Br* construct was made from an *Abd-Bm* cDNA cutting with appropriate enzymes to delete the first exon and the resulting fragment was cloned in UASp [This construct has already been donated for the experiments performed in [41]]. We also generated a mutant Abd-B where the conserved tryptophan at position 381 was substituted by Alanine (Abd-B*W) and subcloned into pCDNA3.

Fragments of the *ems1.2* enhancer (Figure S2A) were subcloned into *phs43-lacZ* to create the following reporter genes *ems0.9*, *ems0.26*, *ems0.35*, *ems0.3*, *emsFragA*, *emsFragD*, *emsFragE* and *emsFragF*. In the *ems0.35* enhancer we mutated the putative Abd-B sites 1, 2, 4A, 4B and 6 individually or in combination to create the single *ems0.35mut1*, *ems0.35mut2*, *ems0.35mut4A*, *ems0.35mut4B*, *ems0.35mut6* or double mutant *ems0.35mut4A4B* and *ems0.35mut4A6* reporter constructs. Site 1 TCATAAA was mutated to >TCTTCAA, site 2 ATAATGA>ATCCCGA, site 4A TCATAAA>TCGGGAA, site 4B TTTATTT>TTCCCTT and site 6 TCATAAA>TCGGGAA. Constructs were injected in *D. melanogaster* by Bestgene (USA) and the *Drosophila* Consolider-Ingenio 2007 transformation platform (Spain). Four to ten independent inserts were analyzed for each line.

DNA sequence analysis to identify conservation regions and DNA binding sites was performed with the JASPAR and the GENOMATIX programs.

### Electrophoretic mobility shift assays (EMSA)

Complementary oligonucleotides (Table S1) were synthesized (Sigma Aldrich). Radioactively labelled probes were generated by annealing and subsequent end filling with [α-32P]dCTP. The conditions used were similar to those described previously [49], [50]. Briefly, double-stranded, end-labelled DNA (50,000 cpm/binding reaction; 10 nM) was incubated with 2 µl of reticulocyte lysate reaction mixture containing each test protein or 2 µl of the lysate control and 50 mM NaCl, 5 mM EDTA, 0,5 mM DTT, 10 mM Tris-HCl (pH 7.8), 4% glycerol, 1 mM mgCl2, and 1 µg of poly dI-dC as nonspecific competitors, in a final reaction volume of 20 µl.

Experiments designed to detect DNA-protein complex formation were performed with a 30-min incubation at 4°C. Reaction mixtures were run on a 5% polyacrylamide gel to visualize complex formation by retardation of the 32P-labeled target DNA. In some experiments monoclonal anti-AbdB was incubated with aliquots of the reaction mixture for an additional 30 min.

The amount of Hth, Exd and Hth/Exd expressing protein lysate used in the experiments detecting Abd-B DNA binding interference, was 2×, 4× and 8× the quantity of protein lysate expressing Abd-B. In all cases the final amount of protein lysate was the same, using non-expressing lysate to equalize the final volume.

Gel electrophoresis was performed in 0.5× Tris-borate-EDTA buffer as described previously [51]. For each gel shift reaction, a control containing the reticulocyte lysate was used to detect possible DNA binding by endogenous lysate factors. Gel was dry at 80°C in vacuum, exposed to a phosphorimager screen and detected by a typhoon scanner.

### Chromatin immunoprecipitation (ChIP) assay

ChIP was performed using transiently transfected *Drosophila* S2 cells [52]. 10×10^6^ cells were seeded in 10 cm cell culture dish, and transfected one day later with either 5 µg pUASt-Abd-B-HA and 5 µg pAC-GAL4 plasmids or 5 µg empty pUASt and 5 µg pAC-GAL4 plasmids. 1/10 of cells were collected to monitor the protein expression by Western blot. The remaining cells were cross-linked, lysed and sheared to 350–1000 bp as described in [53]. Six microliters of anti-HA antibody (Abcam) was used per 100 µg sheared chromatin, and the immunoprecipitation was performed according to [54].

qRT-PCR was done using primers emsQPCR2for and emsQPCR2rev (Table S1) amplifying inside the *ems0.35* enhancer sequence. The data are represented as recovered percentage from the input in AbdB-HA-transfected cells against GAL4-transfected cells.

### GST pull-down

Exd, Hth and Abd-B GST pull-down assays were performed [55] after cloning the Hth or Exd ORF in pCDNA3 (Invitrogen) and labeled *in vitro* with S^35^ by the TNT T7 Quick Coupled Transcription/Translation System (Promega).

From 1000 ml of bacterial culture expressing either GST (negative control), GST-Abd-B313 (a.a. 313 to 493), GST-Abd-B313ΔHD (lacking HD a.a. 384–445) or GST-AbdB-HD (a.a. 386–446), crude extracts were generated and mixed with 70 mg of glutathione agarose beads. After 5 hr of incubation at 4°C, the beads were washed three times in lysis buffer (50 mM Tris-Cl pH 8, 1 mM EDTA, 100 mM ClNa, PMSF 250 µM, DOC 0.1%, CaCl2 5 mM, lysozime 330 µg/ml, DNasaI 66 µg/ml, Triton X-100 1% and complete protease inhibitor 1× (Roche)), then 30 µg of beads-conjugated protein mixed with 300 µl of binding buffer (10 mM Tris-Cl pH 8, 5 mM EDTA, 0.5% DTT, 1 mM MgCl2, 150 mM ClNa, 0.1 mM PMSF and complete protease inhibitor 1× (Roche)), plus 30 µl of S^35^-labelled protein, and incubated for an additional 4 hr at 4°C. The beads were washed four times with binding buffer. A total of 40 µl of SDS loading buffer was added to the beads, which were boiled, spun, and half of supernatant loaded onto an 8% SDS-polyacrylamide gel. After electrophoresis, the gel was dried and detected by phosphorimager method.

## Supporting Information

Figure S1 Ectopic expression of Abd-Bm and r isoforms in embryos using the Gal4 system. (A) Wild type expression of both Abd-B isoforms in st14 embryos. (B) Ectopic Abd-Bm expression in *arm-Gal4 UAS-AbdBm* embryos. (C) Ectopic Abd-Br expression in *arm-Gal4 UAS-AbdBr* embryos. (D) Expression of Abd-Br with the *nullo-Gal4* line at 25°C weakly induces spiracle structures. (E) The same line as in E but grown at 29°C to increase Gal4 efficiency shows some spiracle induction confirming the weak morphogenetic function of this isoform.
(TIF)

Figure S2 Dissection of the *ems* posterior spiracle enhancer. (A) Scheme showing different constructs tested in this work. Asterisks represent putative Abd-B binding sites in *ems0.35*. (B–D) Spiracle expression driven by the *ems0.9* (B) and the *ems0.35* fragment (C–D) is similar to that in the original *ems1.2* construct. (E) Ectopic activation of *ems0.35* after ectopic expression of Abd-Bm driven with *69B-Gal4*. (F) Lack of expression of *ems0.35* in Abd-B^M1^ null mutants. (G) Abd-B binding of the *ems0.35* region in transfected UAS-Abd-B-HA S2 cells compared to control cells. (I–L) Constructs deleting portions of the *ems0.35* fragment as indicated in panel A result in the complete loss of posterior spiracle expression. Note that in Fragment E (L) deletion of the area around site 1 results in the absence of spiracle expression, while point mutation of Abd-B binding site 1 in *ems0.35* (H) does not affect the posterior spiracle expression of the construct indicating the presence of cofactor or collaborator binding sites in the area. (B,C,F) st14 embryos, (D–E,H–L) st11 embryos. Black arrows point to the site of the posterior spiracle primordium, white arrows in (E) point at two ectopic spiracles.
(TIF)

Figure S3 Sequence conservation of the *ems0.35* posterior spiracle enhancer in twelve *Drosophila* species. Alignment of *D. melanogaster*, *D. simulans*, *D. sechellia*, *D. yakuba*, *D. erecta*, *D. ananassae*, *D. pseudobscura*, *D. persimilis*, *D. wilistoni, D. mojavensis*, *D. virilis* and *D. grimshawi* species. Different shades of blue indicate the degree of conservation with dark blue bases being conserved in all twelve species. Dashes indicate inserts in some of the species analyzed. The consensus is labelled underneath with the *Drosophila melanogaster* Abd-B putative binding sites marked as red boxes and Exd and Hth sites as orange and green boxes. The sequence is presented in six fragments that correspond to the six oligos tested in this work. Putative binding sites in this figure were identified using the JASPAR program.
(TIF)

Table S1 Sequence of oligos used in this work.
(DOC)
