# Supplementary figures and images for: Antagonism Versus Cooperativity with TALE Cofactors at the Base of the Functional Diversification of Hox Protein Function
Source: PLoS Genet. 2013 Feb 7;9(2):e1003252. doi: 10.1371/journal.pgen.1003252 (PMC3567137; doi:10.1371/journal.pgen.1003252)

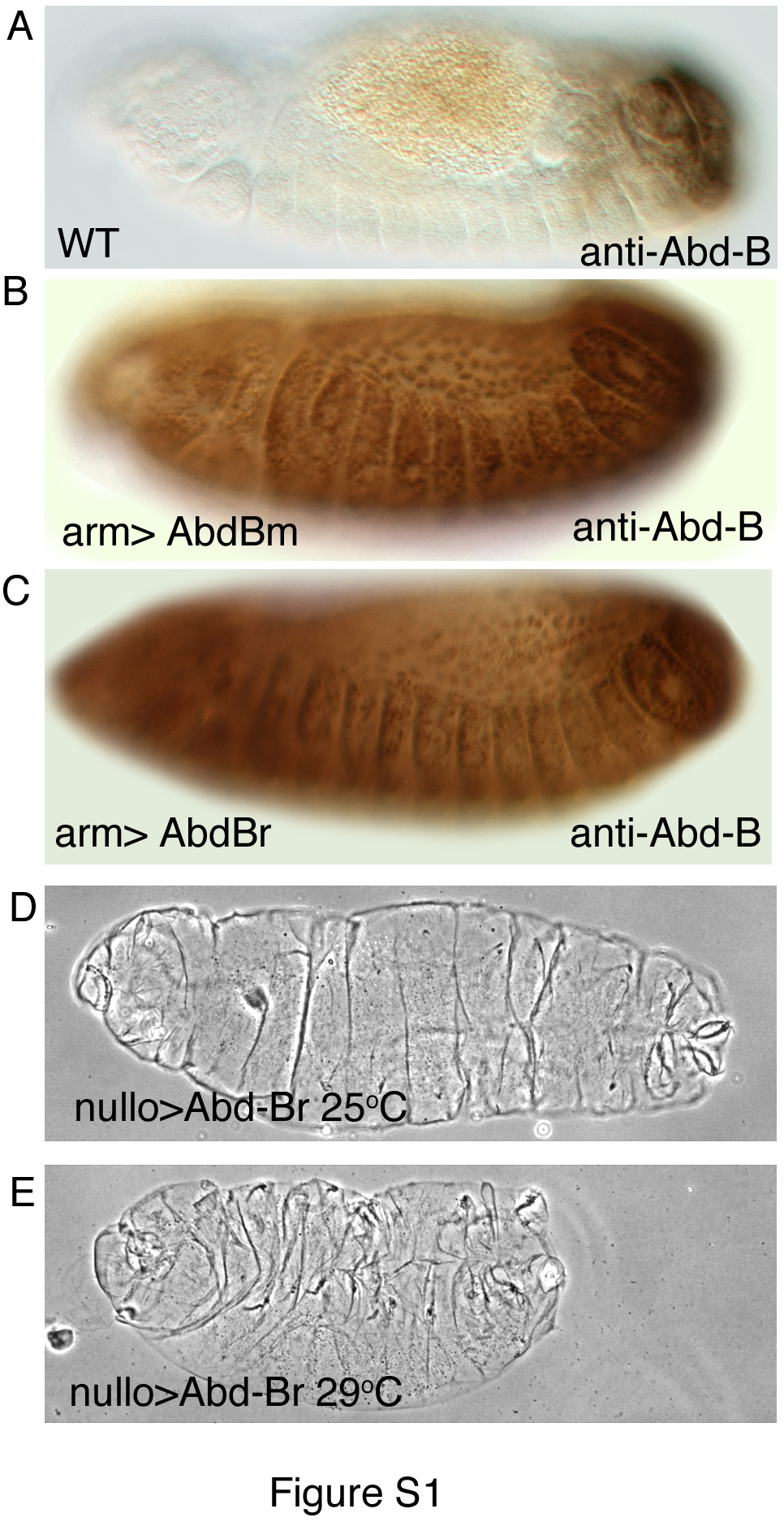

Supplement: Figure S1 — Ectopic expression of Abd-Bm and r isoforms in embryos using the Gal4 system. (A) Wild type expression of both Abd-B isoforms in st14 embryos. (B) Ectopic Abd-Bm expression in arm-Gal4 UAS-AbdBm embryos. (C) Ectopic Abd-Br expression in arm-Gal4 UAS-AbdBr embryos. (D) Expression of Abd-Br with the nullo-Gal4 line at 25°C weakly induces spiracle structures. (E) The same line as in E but grown at 29°C to increase Gal4 efficiency shows some spiracle induction confirming the weak morphogenetic function of this isoform. (TIF) [file pgen.1003252.s001.tif]

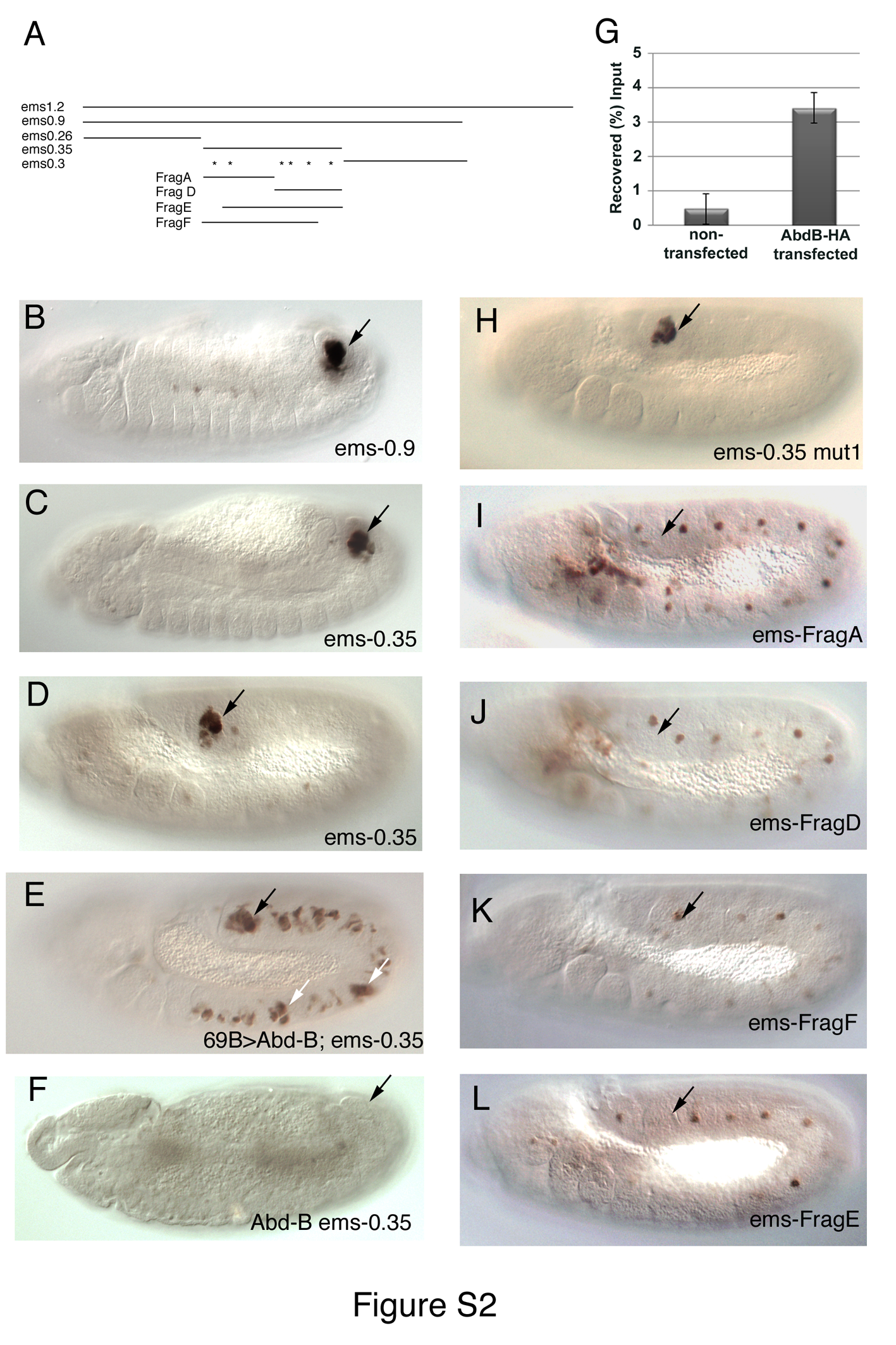

Supplement: Figure S2 — Dissection of the ems posterior spiracle enhancer. (A) Scheme showing different constructs tested in this work. Asterisks represent putative Abd-B binding sites in ems0.35. (B–D) Spiracle expression driven by the ems0.9 (B) and the ems0.35 fragment (C–D) is similar to that in the original ems1.2 construct. (E) Ectopic activation of ems0.35 after ectopic expression of Abd-Bm driven with 69B-Gal4. (F) Lack of expression of ems0.35 in Abd-BM1 null mutants. (G) Abd-B binding of the ems0.35 region in transfected UAS-Abd-B-HA S2 cells compared to control cells. (I–L) Constructs deleting portions of the ems0.35 fragment as indicated in panel A result in the complete loss of posterior spiracle expression. Note that in Fragment E (L) deletion of the area around site 1 results in the absence of spiracle expression, while point mutation of Abd-B binding site 1 in ems0.35 (H) does not affect the posterior spiracle expression of the construct indicating the presence of cofactor or collaborator binding sites in the area. (B,C,F) st14 embryos, (D–E,H–L) st11 embryos. Black arrows point to the site of the posterior spiracle primordium, white arrows in (E) point at two ectopic spiracles. (TIF) [file pgen.1003252.s002.tif]

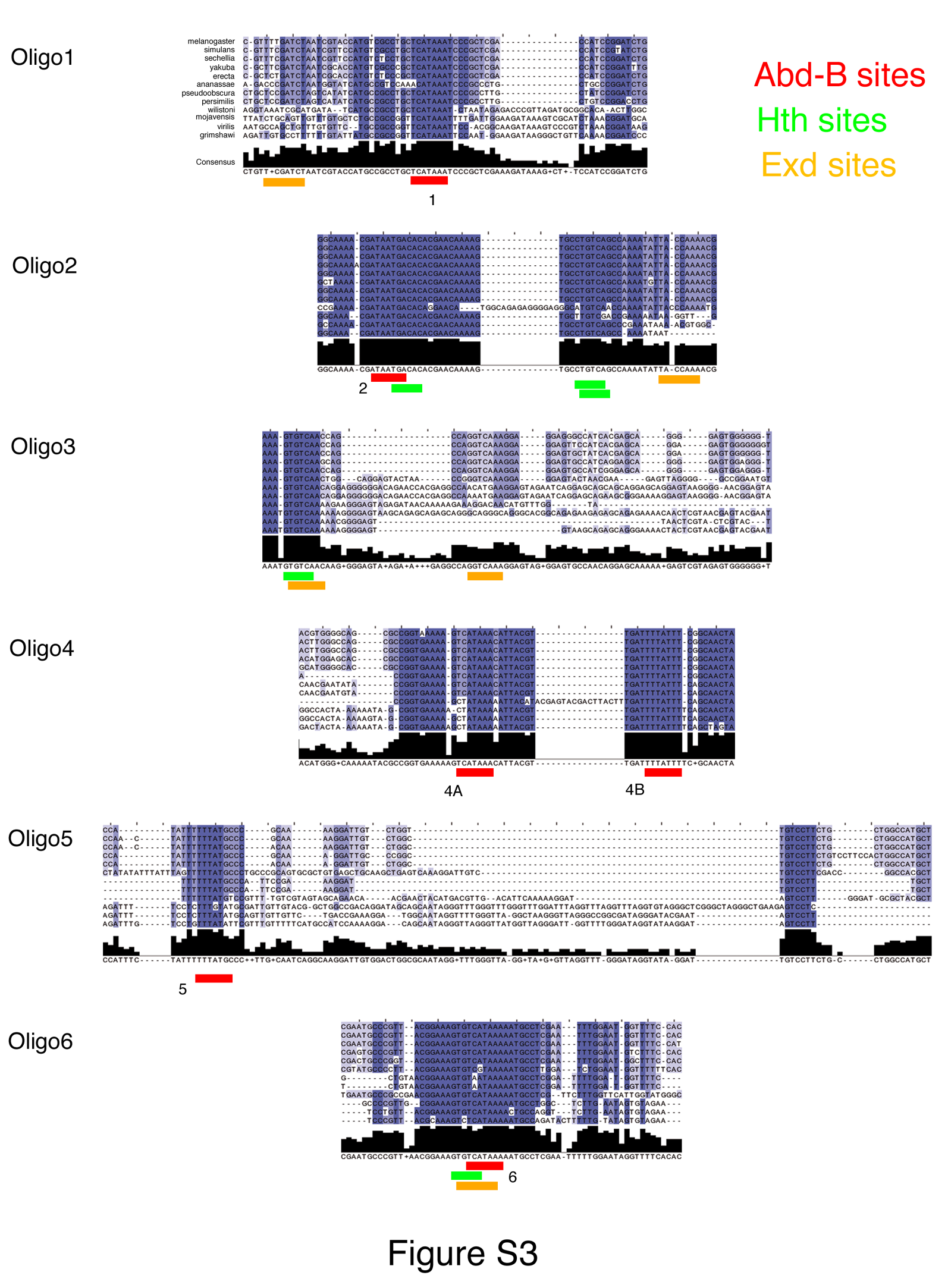

Supplement: Figure S3 — Sequence conservation of the ems0.35 posterior spiracle enhancer in twelve Drosophila species. Alignment of D. melanogaster, D. simulans, D. sechellia, D. yakuba, D. erecta, D. ananassae, D. pseudobscura, D. persimilis, D. wilistoni, D. mojavensis, D. virilis and D. grimshawi species. Different shades of blue indicate the degree of conservation with dark blue bases being conserved in all twelve species. Dashes indicate inserts in some of the species analyzed. The consensus is labelled underneath with the Drosophila melanogaster Abd-B putative binding sites marked as red boxes and Exd and Hth sites as orange and green boxes. The sequence is presented in six fragments that correspond to the six oligos tested in this work. Putative binding sites in this figure were identified using the JASPAR program. (TIF) [file pgen.1003252.s003.tif]
