# Supplementary material for: Antagonism Versus Cooperativity with TALE Cofactors at the Base of the Functional Diversification of Hox Protein Function
Source: PLoS Genet. 2013 Feb 7;9(2):e1003252. doi: 10.1371/journal.pgen.1003252 (PMC3567137; doi:10.1371/journal.pgen.1003252)
Supplement: Table S1 — Sequence of oligos used in this work. (DOC) [file pgen.1003252.s004.doc]

Table S1

| Ems 5' | GAGCTCGGAATCTACTTAGC |
| --- | --- |
| Ems 3' | CTCGAGAAAAATAAGAAAGGG |
| ems 0.26 rev | CGATACAAGGACCTTTGTACAGTG |
| ems 0.30 for | CCACTATTTTCCACTGTTTTGCACACACG |
| ems 0.35 for | CGTTTTGATCTAATCGTACCATGTCGCC |
| ems 0.35 rev | GTGGAAAACCATTCCAAATTCGAGGC |
| AC down | ACCCCCCACTCCCCTGC |
| B down | CGTTTTGGTAATATTTTGGC |
| C up | GGCAAAACGATAATGACACACG |
| D up | ACGTGGGGCAGCGCCGGTAAAAA |
| E down | AGCATGGCCAGCAGAAGGAC |
| G down | TAGTTGCCGAAATAAAATCAACG |
| H up | AAAGTGTCAACCAGCCAGGTC |
| Oli 1 for | tCGTTTTGATCTAATCGTACCATGTCGCCTGCTCATAAATCCCGCTCGACCATCCGGATCTG |
| Oli 1 rev | tCAGATCCGGATGGTCGAGCGGGATTTATGAGCAGGCGACATGGTACGATTAGATCAAAACG |
| Oli 2 for | tGGCAAAACGATAATGACACACGAACAAAAGTGCCTGTCAGCCAAAATATTACCAAAACG |
| Oli 2 rev | tCGTTTTGGTAATATTTTGGCTGACAGGCACTTTTGTTCGTGTGTCATTATCGTTTTGCC |
| Oli 3 for | tAAAGTGTCAACCAGCCAGGTCAAAGGAGGAGGGCCATCACGAGCAGGGGAGTGGGGGGT |
| Oli 3 rev | tACCCCCCACTCCCCTGCTCGTGATGGCCCTCCTCCTTTGACCTGGCTGGTTGACACTTT |
| Oli 4 for | tACGTGGGGCAGCGCCGGTAAAAAGTCATAAACATTACGTTGATTTTATTTCGGCAACTA |
| Oli 4 rev | tTAGTTGCCGAAATAAAATCAACGTAATGTTTATGACTTTTTACCGGCGCTGCCCCACGT |
| Oli 5 for | tCCATATTTTTTATGCCCGCAAAAGGATTGTCTGGTTGTCCTTCTGCTGGCCATGCT |
| Oli 5 rev | tAGCATGGCCAGCAGAAGGACAACCAGACAATCCTTTTGCGGGCATAAAAAATATGG |
| Oli 6 for | tCGAATGCCCGTTACGGAAAGTGTCATAAAAATGCCTCGAATTTGGAATGGTTTTCCA |
| Oli 6 rev | tTGGAAAACCATTCCAAATTCGAGGCATTTTTATGACACTTTCCGTAACGGGCATTCG |
| AbdB site1 for | ATGTCGCCTGctcttcaATCCCGCTCGA |
| AbdB site1 rev | TCGAGCGGGATtgaagagCAGGCGACAT |
| AbdB site2 for | GGCAAAACGAtcccgaCACACGAACAAAAGTG |
| AbdB site2 rev | CACTTTTGTTCGTGTGtcgggaTCGTTTTGCC |
| AbdB site4A for | CGGTAAAAAGtcgggaACATTACGTTGATTttatttCGGC |
| AbdB site4A rev | GCCGaaataaAATCAACGTAATGTtcccgaCTTTTTACCG |
| AbdB site4B for | CGTTGATTtcccttCGGCAACTACCATATTTTTTATGC |
| AbdB site4B rev | GCATAAAAAATATGGTAGTTGCCGaagggaAATCAACG |
| AbdB site6 for | GAAAGTGtcgggaAAATGCCTCGAATTTGGAATGGTTTTC |
| AbdB site6 rev | GAAAACCATTCCAAATTCGAGGCATTTtcccgaCACTTTC |
| Oli 4 for mutA | gACGTGGGGCAGCGCCGGTAAAAAGTCAcccACATTACGTTGATTTTATTTCGGCAACTA |
| Oli 4 rev mutA | gTAGTTGCCGAAATAAAATCAACGTAATGTgggTGACTTTTTACCGGCGCTGCCCCACGT |
| Oli 4 for mutB | gACGTGGGGCAGCGCCGGTAAAAAGTCATAAACATTACGTTGATTcccTTTCGGCAACTA |
| Oli 4 rev mutB | gTAGTTGCCGAAAgggAATCAACGTAATGTTTATGACTTTTTACCGGCGCTGCCCCACGT |
| Oli 4 for mutAB | gACGTGGGGCAGCGCCGGTAAAAAGTCAcccACATTACGTTGATTcccTTTCGGCAACTA |
| Oli 4 rev mutAB | gTAGTTGCCGAAAgggAATCAACGTAATGTgggTGACTTTTTACCGGCGCTGCCCCACGT |
| Oli 6 for mut | gCGAATGCCCGTTACGGAAAGTGTCAcccAAATGCCTCGAATTTGGAATGGTTTTCCA |
| Oli 6 rev mut | gTGGAAAACCATTCCAAATTCGAGGCATTTgggTGACACTTTCCGTAACGGGCATTCG |
| ems QPCR2 for | AGCGCCGGTAAAAAGTCATA |
| ems QPCR2 rev | GCAGAAGGACAACCAGACAA |
